# Inflammaging: disturbed interplay between autophagy and inflammasomes

**DOI:** 10.18632/aging.100444

**Published:** 2012-03-07

**Authors:** Antero Salminen, Kai Kaarniranta, Anu Kauppinen

**Affiliations:** ^1^ Department of Neurology, Institute of Clinical Medicine, University of Eastern Finland, FIN-70211 Kuopio, Finland; ^2^ Department of Neurology, Kuopio University Hospital, FIN-70211 Kuopio, Finland; ^3^ Department of Ophthalmology, Institute of Clinical Medicine, University of Eastern Finland, FIN-70211 Kuopio, Finland; ^4^ Department of Ophthalmology, Kuopio University Hospital, FIN-70211 Kuopio, Finland

**Keywords:** aging, autophagy, inflammasome, inflammation, mitochondria, NF-κB

## Abstract

Inflammaging refers to a low-grade pro-inflammatory phenotype which accompanies aging in mammals. The aging process is associated with a decline in autophagic capacity which impairs cellular housekeeping, leading to protein aggregation and accumulation of dysfunctional mitochondria which provoke reactive oxygen species (ROS) production and oxidative stress. Recent studies have clearly indicated that the ROS production induced by damaged mitochondria can stimulate intracellular danger-sensing multiprotein platforms called inflammasomes. Nod-like receptor 3 (NLRP3) can be activated by many danger signals, e.g. ROS, cathepsin B released from destabilized lysosomes and aggregated proteins, all of which evoke cellular stress and are involved in the aging process. NLRP3 activation is also enhanced in many age-related diseases, e.g. atherosclerosis, obesity and type 2 diabetes. NLRP3 activates inflammatory caspases, mostly caspase-1, which cleave the inactive precursors of IL-1β and IL-18 and stimulate their secretion. Consequently, these cytokines provoke inflammatory responses and accelerate the aging process by inhibiting autophagy. In conclusion, inhibition of autophagic capacity with aging generates the inflammaging condition via the activation of inflammasomes, in particular NLRP3. We will provide here a perspective on the current research of the ROS-dependent activation of inflammasomes triggered by the decline in autophagic cleansing of dysfunctional mitochondria.

## Inflammaging

In 2000, Franceschi et al. [1] coined the term “inflammaging” in order to refer to a low-grade pro-inflammatory status appearing during the aging process. They emphasized the role of macrophages as well as cellular stress and genetic factors in the generation of the inflammaging condition. In addition, they hypothesized that this inflammatory environment could predispose the organism to the development of several age-related diseases. During recent years, this scenario has been confirmed by a plethora of experimental evidence. However, it seems that concurrently with the chronic, low-level inflammation one encounters several symptoms of immunosenescence, both in the innate and adaptive immune systems [2,3]. The presence of a pro-inflammatory phenotype in aged mammals is evident by (i) increased expression of genes linked to inflammation and immune responses in the tissues of old humans and rodents [4-6], (ii) higher level of cytokines in serum, e.g. IL-6 and TNF-α [7,8], (iii) activation of NF-κB signaling which is the master regulator of inflammatory responses [9-11]. There are tissue specific differences in the production of age-related inflammatory factors as well as in the onset and level of pathological changes [12].

It is known that systemic inflammation linked to inflammaging aggravates e.g. the vascular pathology and provokes atherosclerosis [4]. Moreover, increased systemic cytokine levels activate the hypothalamus-pituitary-adrenal (HPA) axis which augments the sercetion of cortisol [13]. Cortisol is a potent anti-inflammatory agent although it not only induces protein catabolism, e.g. in muscle tissues, but it also promotes bone resorption. Chronic inflammation can also enhance the appearance of insulin resistance in muscles and adipose tissues as well as disturb the maintenance of energy homeostasis and subsequently cellular housekeeping functions. Interestingly, the aging process is simultaneously accompanied by both the features accelerating inflammaging and the counteracting, so-called anti-inflammaging characteristics [14]. It seems that the balance between these opposite forces controls the outcome of the aging process, either leading to frailty and degenerative diseases or a healthy old age and longevity.

## Inflammasomes: molecular platforms for danger signal recognition

The aging process jeopardizes the maintenance of cellular homeostasis leading to the activation of a variety of host defence systems. Inflammasomes are intracellular multiprotein sensors which can recognize a large set of danger signals, induced either by pathogens or cellular stress, and once activated, they subsequently stimulate inflammatory responses [15-18]. There are several subfamilies of NOD-like receptors (NLR) but emerging data indicates that the NLRP subfamily, in particular the NLRP3 member, is the major sensor for “intracellular danger-associated molecular patterns” (DAMPs). Inflammasomes are signaling platforms which are assembled after the recognition of DAMP by the receptor protein. In the case of NLRP3, the activated receptor interacts with the adaptor protein ASC which recruits the inflammatory caspase-1 (CASP-1) to the complex which subsequently oligomerizes into penta- or heptameric inflammasomes [16,19]. CASP-1 is the common effector molecule in inflammasomes which cleaves the inactive precursors of two proinflammatory cytokines, i.e. IL-1β and IL-18, into their mature forms which are then secreted from cells. In addition to CASP-1, some other inflammatory caspases, e.g. CASP-4, CASP-5 and CASP-12, can also process the proforms of these cytokine [16,20]. In general, the expression levels of NLRP3 receptor as well as the precursors of IL-1β and IL-18 remain low and activation of NLRP3 inflammasomes requires a priming phase while the expression of these proteins is clearly induced [17,21,22]. Interestingly, NF-κB signaling is a crucial inducer of NLRP3 expression [21]. It should be noted that different cellular stresses and the aging process can stimulate NF-κB signaling [11,23] and probably enhance the priming and potentiation of the inflammasome activation.

Infections and tissue injuries can trigger an inflammatory reaction as a physiological host defence mechanism but in addition, cellular stress can also alert the immune system and induce adaptive responses. This kind of inflammation was termed “para-inflammation” by Medzhitov [24]. Recent studies have revealed that NLRP3 is the major receptor for endogenous danger insults to facilitate inflammatory responses. Petrilli et al. [25] demonstrated that the efflux of potassium, an effect evoked by many noxious stimuli, could activate NLRP3 inflammasomes. After this initial observation, several different activation mechanisms have been identified but still there is an ongoing debate about whether they are physiological inducers and what kind of responses they stimulate. The increased level of reactive oxygen species (ROS) induced by oxidative stress was one of the first stimuli which was demonstrated to trigger NLRP3 activation and promote CASP-1-dependent IL-1β secretion [26,27]. Furthermore, several studies have indicated that the release of cathepsin B after lysosomal damage can activate NLRP3 [28,29]. Lysosomal destabilization is also associated with the NLRP3 activation induced by cholesterol crystals in macrophages [30], probably involved in the inflammation promoting atherosclerosis. There are some observations that amyloid fibrils, e.g. islet amyloid polypeptides (IAPP) and Alzheimer×s amyloid-β, can trigger NLRP3 inflammasomes [28,31] and in that way stimulate inflammation and enhance pathogenesis in type 2 diabetes and Alzheimer×s disease, respectively.

Recently, Wen et al. [32] demonstrated in macrophages that palmitate, a saturated fatty acid, could activate NLRP3 whereas the unsaturated oleate was not responsive. Interestingly, NLRP3 activation was ROS-dependent and secreted IL-1β impaired insulin signaling and promoted insulin resistance in mice. Vandanmagsar et al. [33] revealed that obesity was associated with the activation of NLRP3 in adipose tissue. These workers observed that the weight loss of obese humans, induced by caloric restriction and exercise, was associated with (i) a reduction of NLRP3 expression in adipose tissue, (ii) a decrease in the level of inflammation and (iii) an increase in insulin sensitivity. Supporting these results, Stienstra et al. [34] observed that transgenic mice which lack *Nlrp3*, *Asc* and *caspase-1*genes were resistant to the obesity induced by high-fat diet and protected from insulin resistance. It seems that NLRP3 could be a sensor for metabolic stress recognizing ROS production [35]. Tschopp and Schroder [36] speculated that different danger signal pathways converge and activate NLRP3 inflammasomes via ROS production.

**Figure 1:**
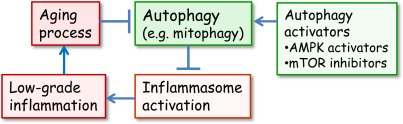
The interplay between autophagy and inflammasomes in the generation of inflammaging Normally, the autophagic uptake of dysfunctional mitochondria prevents the excessive ROS production and in that way the activation of inflammasomes. However, during aging, the autophagic capacity declines and increased ROS production and aggregated proteins activate inflammasomes which provoke a low-grade inflammation in several tissues and in that way inhibit autophagy and accelerate the aging process. There are several activators of autophagy which can delay the aging process. It is known that mTOR inhibitors and AMPK activators can extend lifespan in certain conditions.

Recently, Zhou et al. [37] demonstrated that mitochondria were crucially involved in the activation of NLRP3. They observed that increased mitochondrial production of ROS stimulated NLRP3 whereas the inhibition of ROS production, either with an enhanced autophagic uptake of damaged mitochondria or by inhibiting the expression of voltage-dependent anion channels (VDAC2), significantly suppressed the stimulation of NLRP3. Their study emphasized the significant role of mitochondria and in particular, their proper clearance by autophagy in the regulation of NLRP3 activation. Autophagy was also shown to be a fundamental host defence mechanism against invading intracellular microbes [38]. Moreover, deficiency in autophagy, e.g. in Crohn×s disease, triggers inflammatory responses and leads to tissue injuries [39]. It seems plausible that autophagy, a guardian of cellular sanctity, is a potent anti-inflammatory mechanism which controls the activation of danger sensors, i.e. NLRP3 inflammasomes (Figure 1).

## Autophagy: master of housekeeping prevents inflammasome activation

Autophagy is an ancient housekeeping mechanism which controls the cellular homeostasis by facilitating the removal of misfolded proteins and dysfunctional organelles, e.g. mitochondria and endoplasmic reticulum, for degradation in lysosomal system [39,40]. There are three pathways which can deliver cytoplasmic material for autophagic degradation, i.e. macro- and microautophagy as well as chaperone-mediated autophagy. Macroautophagy, segregating organelles like mitochondria, is the major type of autophagy associated with innate immunity [41,42] and it is hereafter shortly called “autophagy”. In addition to the cleansing function, autophagy can regulate cellular energy balance, e.g. during starvation it can trigger energy production from its own components [43]. Autophagy may also be involved in lipid metabolism by sequestering lipid droplets [44]. In conjunction with this increased knowledge on inflammasomes, the role of autophagy in the regulation of inflammatory responses has started to emerge.

Several studies have clearly indicated that autophagy can suppress inflammatory reactions [42,45-47]. For instance, loss of autophagy proteins, e.g. Atg16L1, potentiates endotoxin-induced IL-1β production [48]. Moreover, genetic studies have revealed that two autophagy genes, *Atg16L1* and *IRGM* are associated with the pathogenesis of Crohn×s disease, an inflammatory bowel disease [39]. It is known that autophagy regulates the inflammatory reaction, e.g. in adipocytes [49] and keratinocytes [50]. Meng and Cai [51] demonstrated that defective autophagy in hypothalamus induced inflammation and subsequently led to obesity and insulin resistance when mice were fed a high-fat diet. Interestingly, these workers observed that the effects of reduced autophagy were reversed by the inhibition of inhibitory-κB kinase β (IKKβ) indicating that inflammation was induced by NF-κB signaling. On the other hand, potentiation of autophagy, e.g. by inhibitors of mammalian target of rapamycin (mTOR) and activators of AMP-activated protein kinase (AMPK), can reduce inflammation and tissue pathology in several diseases [39,45,52] (see also below). Shi et al. [53] demonstrated in human macrophages that increasing the autophagy by starvation and rapamycin treatment reduced CASP-1 activity and secretion of IL-1β whereas blocking the autophagy clearly enhanced inflammasome activity. They also observed that autophagic adaptor protein, p62/sequestosome-1, delivered ubiquitinated inflammasomes to degradation in autophagosomes.

Mitochondria have a crucial role in the regulation of innate immunity responses [54,55]. In addition to the ROS-dependent activation of NLRP3 inflammasomes, mitochondria (i) are involved in the control of antiviral RIG-1-like receptor (RLR) signaling pathways, (ii) contain NLRX1 receptors which monitor e.g. ROS production, and (iii) secrete several DAMPs, such as ROS, mitochondrial DNA (mtDNA) and formyl peptides [54,56]. The studies by Zhou et al. [37] and Nakahira et al. [57] clearly demonstrated that secretion of ROS and mtDNA from mitochondria activated inflammasomes, i.e. mitochondria with disrupted integrity and impaired autophagic clearance are the crucial regulators of inflammasomal activation and subsequently inflammatory responses. Nakahira et al. [57] revealed that depletion of autophagic proteins impaired mitochondrial integrity and increased their ROS production. They also demonstrated that ROS were required for caspase-1 activation, a prerequisite for the maturation of IL-1β and IL-18. They also observed that NLRP3 mediates the release of mtDNA which seems to function as a co-activator of caspase-1. In contrast, Zhou et al. [27] reported that ROS could dissociate the complex between thioredoxin (TRX) and thioredoxin-interacting protein (TXNIP), and consequently TXNIP activated NLRP3. Xiang et al. [58] demonstrated in endothelial cells that ROS produced by NADPH oxidase stimulated the release of TXNIP and its binding to NLRP3 and subsequently induced IL-1β secretion. There is also the possibility that ROS could directly oxidize thiol groups in leucine-rich repeat (LRR) domain of NLRP3 and in that way activate the inflammasomal pathway [17]. Interestingly, there are several studies which demonstrate that ROS can activate autophagy and thus enhance the autophagic cleansing of dysfunctional mitochondria or misfolded proteins [59] and in that way reduce the activation of inflammasomes and the risk for tissue injuries.

In conclusion, all these observations emphasize that a deficiency in the cellular housekeeping can trigger the inflammatory danger sensor NLRP3, and also NLRP1 in some tissues like brain [60], and by this means stimulate inflammatory reactions in sensitive tissues. In this respect, the effective function of autophagic uptake and lysosomal degradation of dysfunctional mitochondria and aggregated proteins is a crucial element in maintaining tissue homeostasis. There are indications that autophagic capacity is compromised in certain diseases [61,62], e.g. in Alzheimer×s disease [63]. On the other hand, there is growing evidence implying that inflammasomes are activated in many pathological conditions [64,65] and thus a deficiency in autophagic housekeeping could trigger an inflammatory component and aggravate their pathogenesis.

## Autophagy declines with aging enhancing the inflammaging process

The aging process involves a progressive decline in cellular and organismal function. The major hallmark of aging is the deficient maintenance of proteostasis which permits the accumulation of damaged and defective cellular components, e.g. lipofuscin, within cells. Brunk and Terman [66] called this cellular status as “garbage can” hypothesis of aging. They proposed that lipofuscin accumulation would disturb lysosomal degradation thus inhibiting the cleansing of dysfunctional mitochondria. After ten years of experimental work, this hypothesis still seems to be valid since different research approaches have demonstrated that autophagy clearly declines with aging and the number of dysfunctional mitochondria augments. In particular, defects in mitochondrial uptake and degradation could increase ROS production and stimulate inflammasomes. Recently, this research topic has been extensively reviewed in detail elsewhere [67-72].

Many studies have indicated that the inhibition of autophagy by genetic manipulation provokes age-related pathological changes and reduces the lifespan of many organisms, e.g. *C. elegans*, *Drosophila* and mice [71,73,74]. Wu et al. [75] demonstrated that the deletion of the *Atg7* gene induced mitochondrial disturbances, e.g. increased production of ROS and alterations in the metabolic profile, in mouse skeletal muscle. Cuervo and Dice [76] observed that also chaperone-mediated autophagy (CMA), not only macroautophagy, was impaired in rat liver during aging. They revealed that the decline was caused by an age-related decrease in the expression of LAMP-2A, the receptor protein of CMA. Interestingly, in subsequent studies [77], they demonstrated that the prevention of the age-related loss of LAMP-2A protein in transgenic mice could maintain efficient CMA and that this was associated with a reduction in the level of damaged proteins and improved liver function during aging. Many studies have also demonstrated that increasing the autophagic capacity by pharmacological or genetical manipulations can prevent the pathology linked to the aging process and even extend lifespan [78-80]. For instance, pharmacological induction of autophagy, e.g. by inhibiting mTOR by rapamycin can increase the lifespan of mice [79] (see below).

Caloric restriction (CR) is the most common anti-aging intervention which can extend lifespan in organisms ranging from yeast to humans [81]. CR is a potent physiological activator of autophagy [82,83]. Several studies have demonstrated that autophagy is required for the CR-mediated lifespan extension [71,83]. Colman et al. [84] revealed that CR delayed the onset of many age-related pathologies, e.g. incidence of diabetes, cancer, cardiovascular disease and brain atrophy, as well as reducing the mortality of rhesus monkeys. Moreover, it is known that CR effectively protects against inflammatory responses and can combat age-related pro-inflammatory phenotype [85,86]. CR has also several beneficial effects on mitochondrial quality control, e.g. ROS production [87,88], which can improve cellular housekeeping and prevent inflammasomal activation. Many studies have indicated that mitochondrial quality control has a key role in the regulation of the aging process [89,90]. For instance, Trifunovic et al. [91] demonstrated that triggering of point mutations and deletions into mtDNA induced a premature aging phenotype in mice.

There are several regulatory mechanisms which could augment the appearance of inflammaging phenotype during the age-related deficiency of autophagy. It is well-known that increased levels of ROS can activate NF-κB signaling via multiple mechanisms [92-94]. For instance, several redox-sensitive protein kinases and phosphatases can stimulate IKK-NF-κB signaling and thus induce and maintain an elevated priming state of inflammasomal system. Moreover, a decline in autophagy can stimulate the activating kinases of the NF-κB complex, i.e. IκB kinase α and β (IKKα/β) and NF-κB-inducing kinase (NIK), which are degraded via selective autophagy [95-97]. Sequestosome-1/p62 is a cargo receptor for selective autophagy [98] and changes in autophagy regulate the p62 level which controls the formation of protein aggregates as well as activation of NF-κB signaling [99,100]. Both of these responses are typical hallmarks of inflammaging and inducers of inflammasomes.

In conclusion, the decline in autophagy during aging creates problems in cellular housekeeping functions which stimulate NF-κB signaling in order to directly or via inflammasomes trigger an age-related pro-inflammatory phenotype. Moreover, there are indications that inflammatory signaling can repress autophagy and thus induce this destructive interplay between autophagy and inflammasomes. For instance, tumor necrosis factor-α (TNF-α), an inflammatory cytokine, can induce or repress autophagy in an NF-κB-dependent manner [101]. In the presence of NF-κB signaling, TNF-α activates mTOR, a major autophagy inhibitor, whereas in cells lacking NF-κB activation, TNF-α treatment stimulates the expression of Beclin 1, an enhancer of autophagy. Both of these responses are dependent on the TNF-α-induced ROS production. Moreover, inflammaging can also enhance inflammasome activation without autophagy since e.g. glucocorticoids, known as anti-inflammatory compounds secreted with aging (see above), can strongly induce the expression of NLRP3 in macrophages [102]. Thus, it seems that the elevated cortisol level as an anti-inflammaging response could increase the priming state of inflammasomes and thus potentiate their response sensitivity during aging.

## Activators of autophagy: promising drugs for inflammaging and related diseases

As Cuervo [67] outlined, the role of autophagy is to “keep that old broom working” with aging. As discussed above, there is much evidence that caloric restriction can activate autophagy and also extend lifespan, probably via its beneficial health effects. Currently, there are a number of drug discovery programs aimed at finding safe chemicals which would be able to activate autophagy. There are two potential groups of compounds acting in this way; mTOR inhibitors [103,104] and AMPK activators [105,106] (Figure 1). mTOR is the major inhibitor of autophagy and is involved in aging [107,108]. Rapamycin and other mTOR inhibitors are either used or under investigation for therapy of cancer and many age-related diseases [107-110]. mTOR is a key protein kinase which couples nutritional and growth factor signaling to protein synthesis, transcription and critical responses in cellular growth, proliferation and survival. Moreover, many studies have reported that lifespan extension induced by CR is associated with the down-regulation of mTOR activity [108,111]. Harrison et al. [79] observed that rapamycin, a recognized inhibitor of mTOR, could extend lifespan in mice if fed late in their life. Recently, Anisimov et al. [112] demonstrated that the lifelong administration of rapamycin extended lifespan in inbred female mice. Moreover, rapamycin could also increase maximal lifespan in cancer-prone mice [113]. Cao et al. [114] observed that rapamycin treatment reversed the senescent phenotype of Hutchinson-Gilford progeria cells in culture by stimulating autophagy. Rapamycin treatment has also been reported to be able to reduce age-related cognitive defects. Majumder et al. [115] demonstrated that life-long administration of rapamycin improved the spatial learning and memory performance in aging mice. Interestingly, this was associated with a decrease in the brain level of IL-1β but not that of TNF-α which could be interpreted to imply that the inflammasomal activity had been reduced by rapamycin therapy. This suggestion is in agreement with the results of Mawhinney et al. [116] which indicated that increased inflammasome activation was linked to age-related cognitive impairment in rats. Rapamycin, also called sirolimus in clinical use, has been intensively studied as an anti-cancer agent [109]. Rapamycin is a macrolide antibiotic and powerful immunosuppressant which causes some serious complications, e.g. lung toxicity. Currently, there are intensive drug discovery programs planned at developing rapalogues, i.e. rapamycin analogues, such as everolimus and temsirolimus [104].

The utilization of AMPK activators is another strategy with which to stimulate autophagy for therapeutic purposes. AMPK is an evolutionary conserved sensor for disturbances in cellular energy balance and a major inducer of autophagy [117,118]. We have recently reviewed the integrated signaling network through which AMPK regulates the aging process [119]. For instance, AMPK can activate autophagy by directly targeting ULK1 which triggers mitophagy [120]. Moreover, AMPK can inhibit the activity of the mTOR complex 1 (mTORC1), either phosphorylating the regulatory Raptor component or activating TSC2 which subsequently inhibit mTOR activity [118]. Interestingly, several studies have indicated that the responsiveness of AMPK signaling declines with aging [118] which could impair autophagic responses during aging. For instance, Reznick et al. [121] observed that physical exercise and AICAR, a chemical activator of AMPK, induced a robust increase in AMPKα2 activity in the skeletal muscles of young mice whereas in old rats no stimulation was apparent. In addition, Liu et al. [122] demonstrated in a mouse model that stroke induced a major activation of AMPK in young animals but no response occurred in their older counterparts. These studies and many other observations indicate that the aging process impairs the activation capacity of AMPK signaling and in that way, disturbs autophagic activity, evokes oxidative stress and triggers the activation of inflammasomes. Moreover, AMPK activity represses NF-κB signaling [123] which could suppress the priming of inflammasomes in young animals but this effect may be lost during aging. There are several pharmacological activators of AMPK, e.g. AICAR (5-aminoimidazole-4-carboxamide ribonucleoside) and the clinically-used antidiabetic drug metformin [105]. Both of these compounds also have AMPK-independent effects. AICAR stimulates AMPK-dependent autophagy via ULK1 and FoxO3a [124,125]. Several natural products, e.g. berberine, curcumin and quercetin, have been reported to activate AMPK signaling [105].

In conclusion, there is substantial evidence indicating that autophagy is a significant regulator of innate immunity responses in host defence. The decline in autophagy with aging impairs cellular housekeeping and exposes cells to the risk of inflammasome activation. There is now convincing experimental data demonstrating that efficient autophagic activity can prevent the activation of inflammasomes and induction of inflammatory responses. AMPK activators and mTOR inhibitors are promising pharmacological agents which can effectively stimulate autophagic degradation. However, many of the present agents have toxic side effects and they can trigger apoptotic cell death. Moreover, it is still a matter of debate whether excessive autophagy can lead to autophagic cell death or not [126]. It seems that generally autophagy is a cell survival mechanism but it can be used for cell death e.g. during development [127] and in some contexts such as in tumor cell death after chemotherapeutic drug treatment [128].

## References

[R1] Franceschi C, Bonafe M, Valensin S, Olivieri F, De Luca M, Ottaviani E, De Benedictis G (2000). Inflamm-aging. An evolutionary perspective on immunosenescence. Ann. NY. Acad. Sci.

[R2] Larbi A, Franceschi C, Mazzatti D, Solana R, Wikby A, Pawelec G (2008). Aging of the immune system as a prognostic factor for human longevity. Physiology.

[R3] Kovacs EJ, Palmer JL, Fortin CF, Fulop T, Goldstein DR, Linton PJ (2009). Aging and innate immunity in the mouse: impact of intrinsic and extrinsic factors. Trends Immunol.

[R4] Csiszar A, Ungvari Z, Koller A, Edwards JG, Kaley G Aging-induced proinflammatory shift in cytokine expression profile in rat coronary arteries. FASEB J.

[R5] de Magalhaes JP, Curado J, Church GM (2009). Meta-analysis of age-related gene expression profiles identifies common signatures of aging. Bioinformatics.

[R6] Swindell WR (2009). Genes and gene expression modules associated with caloric restriction and aging in the laboratory mouse. BMC Genomics.

[R7] Krabbe KS, Pedersen M, Bruunsgaard H (2004). Inflammatory mediators in the elderly. Exp. Gerontol.

[R8] Singh T, Newman AB (2010). Inflammatory markers in population studies of aging. Ageing Res. Rev.

[R9] Helenius M, Hänninen M, Lehtinen SK, Salminen A (1996). Changes associated with aging and replicative senescence in the regulation of transcription factor nuclear factor-κB. Biochem. J.

[R10] Adler AS, Sinha S, Kawahara TL, Zhang JY, Segal E, Chang HY (2007). Motif module map reveals enforcement of aging by continual NF-κB activity. Genes Dev.

[R11] Salminen A, Huuskonen J, Ojala J, Kauppinen A, Kaarniranta K, Suuronen T (2008). Activation of innate immunity system during aging: NF-κB signaling is the culprit of inflamm-aging. Ageing Res. Rev.

[R12] Cevenini E, Caruso C, Candore G, Capri M, Nuzzo D, Duro G, Rizzo C, Colonna-Romano G, Lio D, Di Carlo D, Palmas MG, Scurti M, Pini E, Franceschi C, Vasto S (2010). Age-related inflammation: the contribution of different organs, tissues and systems. How to face it for therapeutic approaches. Curr. Pharm. Des.

[R13] Giunta G (2008). Exploring the complex relations between inflammation and aging (inflamm-aging): anti-inflamm-aging remodelling of inflamm-aging, from robustness to frailty. Inflamm. Res.

[R14] Franceschi C, Capri M, Monti D, Giunta S, Olivieri F, Sevini F, Panourgia MP, Invidia L, Celani L, Scurti M, Cevenini E, Castellani GC, Salvioli S (2007). Inflammaging and anti-inflammaging: a systemic perspective on aging and longevity emerged from studies in humans. Mech. Age. Dev.

[R15] Chen G, Shaw MH, Kim YG, Nunez G (2009). NOD-like receptors: role in innate immunity and inflammatory disease. Annu. Rev. Pathol. Mech. Dis.

[R16] Schroder K, Tschopp J (2010). The inflammasomes. Cell.

[R17] Gross O, Thomas CJ, Guarda G, Tschopp J (2011). The inflammasome: an integrated view. Immunol. Rev.

[R18] Kersse K, Bertrand MJM, Lamkanfi M, Vandenabeele P (2011). NOD-like receptors and the innate immune system: coping with danger, damage and death. Cytokine Growth Factor Rev.

[R19] Martinon F, Tschopp J (2007). Inflammatory caspases and inflammasomes: master switches of inflammation. Cell Death Differ.

[R20] Yazdi AS, Guarda G, D'Ombrain MC, Drexler SK (2010). Inflammatory caspases in innate immunity and inflammation. J. Innate Immun.

[R21] Bauernfeind F, Ablasser A, Bartok E, Kim S, Schmid-Burgk J, Cavlar T, Hornung V (2011). Inflammasomes: current understanding and open questions. Cell. Mol. Life Sci.

[R22] Bauernfeind FG, Horvath G, Stutz A, Alnemri ES, MacDonald K, Speert D, Fernandes-Alnemri T, Wu J, Monks BG, Fitzgerald KA, Hornung V, Latz E (2009). Cutting edge: NF-κB activating pattern recognition and cytokine receptors license NLRP3 inflammasome activation by regulating NLRP3 expression. J. Immunol.

[R23] Piva R, Belardo G, Santoro MG (2006). NF-κB: a stress-regulated switch for cell survival. Antioxid. Redox Signal.

[R24] Medzhitov R (2008). Origin and physiological roles of inflammation. Nature.

[R25] Petrilli V, Papin S, Dostert C, Mayor A, Martinon F, Tschopp J (2007). Activation of the NALP3 inflammasome is triggered by low intracellular potassium concentration. Cell Death Differ.

[R26] Cruz CM, Rinna A, Forman HJ, Ventura ALM, Persechini PM, Ojcius DM (2007). ATP activates a reactive oxygen species-dependent oxidative stress response and secretion of proinflammatory cytokines in macrophages. J. Biol. Chem.

[R27] Zhou R, Tardivel A, Thorens B, Choi I, Tschopp J (2010). Thioredoxin-interacting protein links oxidative stress to inflammasome activation. Nat. Immunol.

[R28] Halle A, Hornung V, Petzold GC, Stewart CR, Monks BG, Reinheckel T, Fitzgerald KA, Latz E, Moore KJ, Golenbock DT (2008). The NALP3 inflammasome is involved in the innate immune response to amyloid-ß. Nature Immunol.

[R29] Niemi K, Teirilä L, Lappalainen J, Rajamäki K, Baumann MH, Öörni K, Wolff H, Kovanen PT, Matikainen S, Eklund KK (2011). Serum amyloid A activates the NLRP3 inflammasome via P2X7 receptor and cathepsin B-sensitive pathway. J. Immunol.

[R30] Rajamäki K, Lappalainen J, Öörni K, Välimäki E, Matikainen S, Kovanen PT, Eklund KK (2010). Cholesterol crystals activate the NLRP3 inflammasome in human macrophages: a novel link between cholesterol metabolism and inflammation. PLoS One.

[R31] Masters SL, O'Neill LA (2011). Disease-associated amyloid and misfolded protein aggregates activate the inflammasome. Trends Mol. Med.

[R32] Wen H, Gris D, Lei Y, Jha S, Zhang L, Huang MTH, Brickey WJ, Ting JPY (2011). Fatty acid-induced NLRP3-ASC inflammasome activation interferes with insulin signaling. Nat. Immunol.

[R33] Vandanmagsar B, Youm YH, Ravussin A, Galgani JE, Stadler K, Mynatt RL, Ravussin E, Stephens JM, Dixit VD (2011). The NLRP3 inflammasome instigates obesity-induced inflammation and insulin resistance. Nat. Med.

[R34] Stienstra R, van Diepen JA, Tack CJ, Zaki MH, van de Veerdonk FL, Perera D, Neale GA, Hooiveld GJ, Hijmans A, Vroegrijk I, van den Berg S, Romijn J, Rensen PC, Joosten LA, Netea MG, Kanneganti TD (2011). Inflammasome is a central player in the induction of obesity and insulin resistance. Proc. Natl. Acad. Sci. USA.

[R35] Schroder K, Zhou R, Tschopp J (2010). The NLRP3 inflammasome: a sensor for metabolic danger?. Science.

[R36] Tschopp J, Schroder K (2010). NLRP3 inflammasome activation: the convergence of multiple signalling pathways on ROS production?. Nat. Rev. Immunol.

[R37] Zhou R, Yazdi AS, Menu P, Tschopp J (2011). A role for mitochondria in NLRP3 inflammasome activation. Nature.

[R38] Deretic V (2010). Autophagy in infection. Curr. Opin. Cell Biol.

[R39] Ravikumar B, Sarkar S, Davies JE, Futter M, Garcia-Arencibia M, Green-Thompson ZW, Jimenez-Sanchez M, Korolchuk VI, Lichtenberg M, Luo S, Massey CO, Menzies FM, Moreau K, Narayanan U, Renna M, Siddiqi FH, Underwood BR, Winslow AR, Rubinsztein DC (2010). Regulation of mammalian autophagy in physiology and pathophysiology. Physiol. Rev.

[R40] He C, Klionsky DJ (2009). Regulation mechanisms and signaling pathways of autophagy. Annu. Rev. Genet.

[R41] Deretic V (2009). Multiple regulatory and effector roles of autophagy in immunity. Curr. Opin. Immunol.

[R42] Levine B, Mizushima N, Virgin HW (2011). Autophagy in immunity and inflammation. Nature.

[R43] Singh R, Cuervo AM (2011). Autophagy in the cellular energetic balance. Cell Metab.

[R44] Dong H, Czaja MJ (2011). Regulation of lipid droplets by autophagy. Trends Endocrinol. Metab.

[R45] Choi AJS, Ryter SW (2011). Autophagy in inflammatory diseases. Int. J. Cell Biol.

[R46] Fesus L, Demeny MA, Petrovski G (2011). Autophagy shapes inflammation. Antioxid. Redox Signal.

[R47] Jo EK, Shin DM, Choi AMK (2012). Autophagy: cellular defence to excessive inflammation. Microbes Infect.

[R48] Saitoh T, Fujita N, Jang MH, Uematsu S, Yang BG, Satoh T, Omori H, Noda T, Yamamoto N, Komatsu M, Tanaka K, Kawai T, Tsujimura T, Takeuchi O, Yoshimori T, Akira S (2008). Loss of the autophagy protein Atg16L1 enhances endotoxin-induced IL-1β production. Nature.

[R49] Yoshizaki T, Kusunoki C, Kondo M, Yasuda M, Kume S, Morino K, Sekine O, Ugi S, Uzu T, Nishio Y, Kashiwagi A, Maegawa H (2012). Autophagy regulates inflammation in adipocytes. Biochem. Biophys. Res. Commun.

[R50] Lee HM, Shin DM, Yuk JM, Shi G, Choi DK, Lee SH, Huang SM, Kim JM, Kim CD, Lee JH, Jo EK (2011). Autophagy negatively regulates keratinocytes inflammatory responses via scaffolding protein p62/SQSTM1. J. Immunol.

[R51] Meng Q, Cai D (2011). Defective hypothalamic autophagy directs the central pathogenesis of obesity via the IκB kinase β (IKKβ)/NF-κB pathway. J. Biol. Chem.

[R52] Hsieh CH, Pai PY, Hsueh HW, Yuan SS, Hsieh YC (2011). Complete induction of autophagy is essential for cardioprotection in sepsis. Ann. Surg.

[R53] Shi CS, Shenderov K, Huang NN, Kabat J, Abu-Asab M, Fitzgerald KA, Sher A, Kehrl JH (2012). Activation of autophagy by inflammatory signals limits IL-1β production by targeting ubiquitinated inflammasomes for destruction. Nat. Immunol.

[R54] Arnoult D, Soares F, Tattoli I, Girardin SE (2011). Mitochondria in innate immunity. EMBO Rep.

[R55] West AP, Shadel GS, Ghosh S (2011). Mitochondria in innate immune responses. Nat. Rev. Immunol.

[R56] Krysko DV, Agostimis P, Krysko O, Garg AD, Bachert C, Lambrecht BN, Vandenabeele P (2011). Emerging role of damage-associated molecular patterns derived from mitochondria in inflammation. Trends Immunol.

[R57] Nakahira K, Haspel JA, Rathinam VAK, Lee SJ, Dolinay T, Lam HC, Englert JA, Rabinovitch M, Cernadas M, Kim HP, Fitzgerald KA, Ryter SW, Choi AMK (2011). Autophagy proteins regulate innate immune responses by inhibiting the release of mitochondrial DNA mediated by the NALP3 inflammasome. Nat. Immunol.

[R58] Xiang M, Shi X, Li Y, Xu J, Yin L, Xiao G, Scott MJ, Billiar TR, Wilson MA, Fan J (2011). Hemorrhagic shock activation of NLRP3 inflammasome in lung endothelial cells. J. Immunol.

[R59] Scherz-Shouval R, Elazar Z (2011). Regulation of autophagy by ROS: physiology and pathology. Trends Biochem. Sci.

[R60] de Rivero Vaccari JP, Lotocki G, Alonso OF, Bramlett HM, Dietrich WD, Keane RW (2009). Therapeutic neutralization of the NLRP1 inflammasome reduces the innate immune response and improves histopathology after brain injury. J. Cereb. Blood Flow Metab.

[R61] Martinez-Vicente M, Cuervo AM (2007). Autophagy and neurodegeneration: when the cleaning crew goes on strike. Lancet Neurol.

[R62] Levine B, Kroemer G (2008). Autophagy in the pathogenesis of disease. Cell.

[R63] Nixon RA, Yang DS (2011). Autophagy failure in Alzheimer's disease - locating the primary defect. Neurobiol. Dis.

[R64] Davis BK, Wen H, Ting JP (2011). The inflammasome NLRs in immunity, inflammation, and associated diseases. Annu. Rev. Immunol.

[R65] Lamkanfi M, Vande Walle L, Kanneganti TD (2011). Deregulated inflammasome signaling in disease. Immunol. Rev.

[R66] Brunk UT, Terman A (2002). The mitochondrial-lysosomal axis theory of aging. Accumulation of damaged mitochondria as a result of imperfect autophagocytosis. Eur. J. Biochem.

[R67] Cuervo AM (2008). Autophagy and aging: keeping that old broom working. Trends Genet.

[R68] Rajawat YS, Hilioti Z, Bossis I (2009). Aging: Central role for autophagy and the lysosomal degradative system. Ageing Res. Rev.

[R69] Salminen A, Kaarniranta K (2009). Regulation of the aging process by autophagy. Trends Mol. Med.

[R70] Green DR, Galluzzi L, Kroemer G (2011). Mitochondria and the autophagy-inflammation-cell death axis in organismal aging. Science.

[R71] Rubinsztein DC, Marino G, Kroemer G (2011). Autophagy and aging. Cell.

[R72] Rezzani R, Stacchiotti A, Rodella LF (2012). Morphological and biochemical studies on aging and autophagy. Ageing Res. Rev.

[R73] Toth ML, Sigmond T, Borsos E, Barna J, Erdelyi P, Takacs-Vellai K, Orosz L, Kovacs AL, Csikos G, Sass M, Vellai T (2008). Lonevity pathways converge on autophagy genes to regulate life span in *Caenorhabditis elegans*. Autophagy.

[R74] Yang L, Li P, Fu S, Calay ES, Hotamisligil GS (2010). Defective hepatic autophagy in obesity promotes ER stress and causes insulin resistance. Cell Metab.

[R75] Wu JJ, Quijano C, Chen E, Liu H, Cao L, Fergusson MM, Rovira II, Gutkind S, Daniels MP, Komatsu M, Finkel T (2009). Mitochondrial dysfunction and oxidative stress mediate the physiological impairment induced by the disruption of autophagy. Aging (Albany NY).

[R76] Cuervo AM, Dice JF (2000). Age-related decline in chaperone-mediated autophagy. J. Biol. Chem.

[R77] Zhang C, Cuervo AM (2008). Restoration of chaperone-mediated autophagy in aging liver improves cellular maintenance and hepatic function. Nat. Med.

[R78] Simonsen A, Cumming RC, Brech A, Isakson P, Schubert DR, Finley KD (2008). Promoting basal levels of autophagy in the nervous system enhances longevity and oxidant resistance in adult *Drosophila*. Autophagy.

[R79] Harrison DE, Strong R, Sharp ZD, Nelson JF, Astle CM, Flurkey K, Nadon NL, Wilkinson JE, Frenkel K, Carter CS, Pahor M, Javors MA, Fernandez E, Miller RA (2009). Rapamycin fed late in life extends lifespan in genetically heterogeneous mice. Nature.

[R80] Madeo F, Tavernarakis N, Kroemer G (2010). Can autophagy promote longevity?. Nat. Cell Biol.

[R81] Bishop NA, Guarente L (2007). Genetic links between diet and lifespan: shared mechanisms from yeast to humans. Nat. Rev. Genet.

[R82] Bergamini E, Cavallini G, Donati A, Gori Z (2007). The role of autophagy in aging: its essential part in the anti-aging mechanism of caloric restriction. Ann. N.Y. Acad. Sci.

[R83] Jia K, Levine B (2007). Autophagy is required for dietary restriction-mediated life span extension in *C. elegans*. Autophagy.

[R84] Colman TJ, Anderson RM, Johnson SC, Kastman EK, Kosmatka KJ, Beasley TM, Allison DB, Cruzen C, Simmons HA, Kemnitz JW, Weindruch R (2009). Caloric restriction delays disease onset and mortality in rhesus monkeys. Science.

[R85] Holloszy JO, Fontana L (2007). Caloric restriction in humans. Exp. Gerontol.

[R86] Morgan TE, Wong AM, Finch CE (2007). Anti-inflammatory mechanisms of dietary restriction in slowing aging processes. Interdiscip. Top. Gerontol.

[R87] Page MM, Robb EL, Salway KD, Stuart JA (2010). Mitochondrial redox metabolism: aging, longevity and dietary effects. Mech. Ageing Dev.

[R88] Hagopian K, Chen Y, Simmons Domer K, Soo Hoo R, Bentley T, McDonald RB, Ramsey JJ (2011). Caloric restriction influences hydrogen peroxide generation in mitochondrial sub-populations from mouse liver. J. Bioenerg. Biomembr.

[R89] Seo AY, Joseph AM, Dutta D, Hwang JC, Aris JP, Leeuwenburgh C (2010). New insights into the role of mitochondria in aging: mitochondrial dynamics and more. J. Cell Sci.

[R90] Weber TA, Reichert AS (2010). Impaired quality control of mitochondria: aging from a new perspective. Exp. Gerontol.

[R91] Trifunovic A, Wredenberg A, Falkenberg M, Spelbrink JN, Rovio AT, Bruder CE, Bohlooly YM, Gidlof S, Oldfors A, Wibom R, Tornell J, Jacobs HT, Larsson NG (2004). Premature ageing in mice expressing defective mitochondrial DNA polymerase. Nature.

[R92] Gloire G, Legrand-Poels S, Piette J (2006). NF-κB activation by reactive oxygen species: fifteen years later. Biochem. Pharmacol.

[R93] Pantano C, Reynaert NL, Van der Vliet A, Janssen-Heininger YMW (2006). Redox-sensitive kinases of the nuclear factor-κB signaling pathway. Antioxid. Redox Signal.

[R94] Morgan MJ, Liu Z (2011). Crosstalk of reactive oxygen species and NF-κB signaling. Cell Res.

[R95] Qing G, Yan P, Xiao G (2006). Hsp90 inhibition results in autophagy-mediated proteasomes-independent degradation of IκB kinase (IKK). Cell Res.

[R96] Qing G, Yan P, Qu Z, Liu H, Xiao G (2007). Hsp90 regulates processing of NF-κB2 p100 involving protection of NF-κB-inducing kinase (NIK) from autophagy-mediated degradation. Cell Res.

[R97] Niida M, Tanaka M, Kamitani (2010). Downregulation of active IKKβ by Ro52-mediated autophagy. Mol. Immunol.

[R98] Johansen T, Lamark T (2011). Selective autophagy mediated by autophagic adapter proteins. Autophagy.

[R99] Bjorkoy G, Lamark T, Johansen T (2006). p62/SQSTM1: a missing link between protein aggregates and the autophagy machinery. Autophagy.

[R100] Moscat J, Diaz-Meco MT, Wooten MW (2007). Signal integration and diversification through the p62 scaffold protein. Trends Biochem. Sci.

[R101] Djavaheri-Mergny M, Amelotti M, Mathieu J, Besancon F, Bauvy C, Souquere S, Pierron G, Codogno P (2006). NF-κB activation represses tumor necrosis factor-α-induced autophagy. J. Biol. Chem.

[R102] Busillo JM, Azzam KM, Cidlowski JA (2011). Glucocorticoids sensitize the innate immune system through regulation of the NLRP3 inflammasome. J. Biol. Chem.

[R103] Tsang CK, Qi H, Liu LF, Zheng XFS (2007). Targeting mammalian target of rapamycin (mTOR) for health and diseases. Drug Discov. Today.

[R104] Benjamin D, Colombi M, Moroni C, Hall MN (2011). Rapamycin passes the torch: a new generation of mTOR inhibitors. Nat. Rev. Drug Discov.

[R105] Fogarty S, Hardie DG (2010). Development of protein kinase activators: AMPK as a target in metabolic disorders and cancer. Biochim. Biophys. Acta.

[R106] Vingtdeux V, Chandakkar P, Zhao H, d'Abramo C, Davis P, Marambaud P (2011). Novel synthetic small-molecule activators of AMPK as enhancers of autophagy and amyloid-β peptide degradation. FASEB J.

[R107] Blagosklonny MV (2008). Aging. ROS or TOR. Cell Cycle.

[R108] Kapahi P, Chen D, Rogers AN, Katewa SD, Li PW, Thomas EL (2010). With TOR, Less is more: a key role for the conserved nutrient-sensing TOR pathway in Aging. Cell Metab.

[R109] Zoncu R, Efeyan A, Sabatini DM (2011). mTOR: from growth signal integration to cancer, diabetes and ageing. Nat. Rev. Mol. Cell Biol.

[R110] Blagosklonny MV Rapamycin and quasi-programmed aging. Four years later. Cell Cycle.

[R111] Blagosklonny MV (2010). Calorie restriction. Decelerating mTOR-driven aging from cells to organisms (including humans). Cell Cycle.

[R112] Anisimov VN, Zabezhinski MA, Popovich IG, Piskunova TS, Semenchenko AV, Tyndyk ML, Yurova MN, Rosenfeld SV, Blagosklonny MV (2011). Rapamycin increases lifespan and inhibits spontaneous tumorigenesis in inbred female mice. Cell Cycle.

[R113] Anisimov VN, Zabezhinski MA, Popovich IG, Piskunova TS, Semenchenko AV, Tyndyk ML, Yurova MN, Antoch MP, Blagosklonny MV (2010). Rapamycin extends maximal lifespan in cancer-prone mice. Am. J. Pathol.

[R114] Cao K, Graziotto JJ, Blair CD, Mazzulli JR, Erdos MR, Krainc D, Collins FS (2011). Rapamycin reverses cellular phenotypes and enhances mutant protein clearance in Hutchinson-Gilford progeria syndrome cells. Sci. Transl. Med.

[R115] Majumder S, Caccamo A, Medina DX, Benavides AD, Javors MA, Kraig E, Strong R, Richardson A, Oddo S (2012). Life-long rapamycin administration ameliorates age-dependent cognitive deficits by reducing IL-1β and NMDA signaling. Aging Cell.

[R116] Mawhinney LJ, de Vaccari Rivero JP, Dale GA, Keane RW, Bramlett HM (2011). Heightened inflammasome activation is linked to age-related cognitive impairment in Fischer 344 rats. BMC Neurosci.

[R117] Steinberg GR, Kemp BE (2009). AMPK in health and disease. Physiol. Rev.

[R118] Mihaylova MM, Shaw RJ (2011). The AMPK signalling pathway coordinates cell growth, autophagy and metabolism. Nat. Cell Biol.

[R119] Salminen A, Kaarniranta K (2012). AMP-activated protein kinase (AMPK) controls the aging process via an integrated signaling network. Ageing Res. Rev.

[R120] Egan DF, Shackelford DB, Mihaylova MM, Gelino S, Kohnz RA, Mair W, Vasquez DS, Joshi A, Gwinn DM, Taylor R, Asara JM, Fitzpatrick J, Dillin A, Viollet B, Kundu M, Hansen M, Shaw RJ (2011). Phosphorylation of ULK1 (hATG1) by AMP-activated protein kinase connects energy sensing to mitophagy. Science.

[R121] Reznick RM, Zong H, Li J, Morino K, Moore IK, Yu HJ, Liu ZX, Dong J, Mustard KJ, Hawley SA, Befroy D, Pypaert M, Hardie DG, Young LH, Shulman GI (2007). Aging-associated reductions in AMPK-activated protein kinase activity and mitochondrial biogenesis. Cell. Metab.

[R122] Liu F, Benashski SE, Persky R, Xu Y, Li J, McCullough LD (2012). Age-related changes in AMP-activated protein kinase after stroke. Age (Dordr).

[R123] Salminen A, Hyttinen JM, Kaarniranta K (2011). AMP-activated protein kinase inhibits NF-κB signaling and inflammation: impact on healthspan and lifespan. J. Mol. Med. (Berl.).

[R124] Lee JW, Park S, Takahashi Y, Wang HG (2010). The association of AMPK with ULK1 regulates autophagy. PLoS One.

[R125] Sanchez AM, Csibi A, Raibon A, Cornille K, Gay S, Bernardi H, Candau R (2012). AMPK promotes skeletal muscle autophagy through activation of forkhead FoxO3a and interaction with Ulk1. J. Cell. Biochem.

[R126] Kroemer G, Levine B (2008). Autophagic cell death: the story of a misnomer. Nat. Rev. Mol. Cell Biol.

[R127] Shimizu S, Konishi A, Nishida Y, Mizuta T, Nishina H, Yamamoto A, Tsujimoto Y (2010). Involvement of JNK in the regulation of autophagic cell death. Oncogene.

[R128] Notte A, Leclere L, Michiels C (2011). Autophagy as a mediator of chemotherapy-induced cell death in cancer. Biochem. Pharmacol.

